# The Roles of Interleukin-6 in the Pathogenesis of Rheumatoid Arthritis

**DOI:** 10.1155/2011/765624

**Published:** 2011-05-26

**Authors:** Misato Hashizume, Masahiko Mihara

**Affiliations:** Product Research Department, Fuji-Gotemba Research Laboratories, Chugai Pharmaceutical Co., Ltd., 1-135 Komakado, Gotemba, Shizuoka 412-8513, Japan

## Abstract

Several clinical studies have demonstrated that the humanized anti-interleukin-6 (IL-6) receptor antibody tocilizumab (TCZ) improves clinical symptoms and prevents progression of joint destruction in rheumatoid arthritis (RA). However, the precise mechanism by which IL-6 blockade leads to the improvement of RA is not well understood. IL-6 promotes synovitis by inducing neovascularization, infiltration of inflammatory cells, and synovial hyperplasia. IL-6 causes bone resorption by inducing osteoclast formation via the induction of RANKL in synovial cells, and cartilage degeneration by producing matrix metalloproteinases (MMPs) in synovial cells and chondrocytes. Moreover, IL-6 is involved in autoimmunity by altering the balance between T_h_17 cells and T_reg_. IL-6 also acts on changing lipid concentrations in blood and on inducing the production of hepcidin which causes iron-deficient anemia. In conclusion, IL-6 is a major player in the pathogenesis of RA, and current evidence indicates that the blockade of IL-6 is a beneficial therapy for RA patients.

## 1. Introduction

Rheumatoid arthritis (RA) is a chronic, systemic autoimmune inflammatory disorder that may affect many tissues and organs, but principally attacks the synovium of joints. The process induces synovitis (infiltration of inflammatory cells such as macrophages and lymphocytes), synovial hyperplasia with neovascularization, and excess synovial fluid, which causes joint swelling, stiffness, and pain. The final results are the destruction of articular cartilage and the erosion of bone in the joints, with some patients suffering permanent disability. RA patients may develop multiple systemic symptoms including fever, fatigue, anemia, anorexia, osteoporosis, weight loss, and muscle weakness. Patient lifespan is reduced by up to 10 years because of cardiovascular disease resulting from chronic inflammation [1]. If untreated, by 5 years after diagnosis about 40% of patients are unable to work, and by 10 years, over 50% are unable to work. Recently, drug management aims to relieve symptoms, as pain relief is the priority for people with RA, and to modify the disease process.

Although the etiology of RA is not fully understood, it has been demonstrated that IL-6 plays a crucial role in its pathogenesis. In fact, treatment of RA patients with the humanized anti-interleukin-6 receptor (IL-6R) antibody, tocilizumab (TCZ), is highly effective [2, 3]. IL-6 is a multifunctional cytokine with biological activities that include regulation of immune response, inflammation, and hematopoiesis. IL-6 also stimulates the secretory activity of the hypothalamus-pituitary-adrenal gland axis and increases adrenocorticotropic hormone and cortisol. IL-6 possesses several proinflammatory properties, such as stimulating the production of chemokines and adhesion molecules in lymphocytes [4], inducing acute-phase proteins in liver cells [5] and increasing neutrophil counts in the blood [6].

In this paper, we summarize the biological function of IL-6 in RA pathogenesis and the mode of action of TCZ on RA patients based on our and others' recent research. 

## 2. IL-6 Signal Transduction

**Figure 1:**
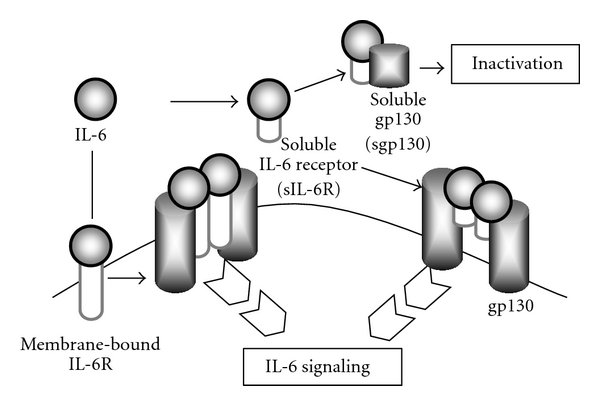
IL-6 signaling.

IL-6 exerts its biological activities through two molecules, a IL-6-specific receptor and a signal transducer, gp130 [9]. When IL-6 binds to membrane-bound IL-6R (mIL-6R), the homodimerization of gp130 is induced, and a high-affinity functional receptor complex of IL-6, IL-6R, and gp130 is formed. On the other hand, the soluble IL-6R (sIL-6R), lacking the intracytoplasmic portion of mIL-6R, is produced either by the enzymatic cleavage of mIL-6R or by alternative splicing. sIL-6R can bind with IL-6 and then the complex of IL-6 and sIL-6R can form the complex with gp130 (Figure 1). This unique receptor signal is termed IL-6 transsignaling [10]. Tocilizumab is able to bind to both sIL-6R and mIL-6R and to inhibit IL-6 binding to its receptors, leading to the blockade of the IL-6 signaling through both receptors [11]. 

Membrane bound gp130 (mgp130) is expressed ubiquitously in the body. Therefore, the IL-6/sIL-6R complex could, theoretically, stimulate most cells of the body. However, this transsignaling is thought to be highly regulated by soluble gp130 (sgp130), which is found at higher concentrations in blood. sgp130 binds IL-6/sIL-6R complex and then inhibits the binding of IL-6/sIL-6R complex to mgp130 [12, 13]. Namely, sgp130 is a natural inhibitor of IL-6 signaling.

 As mentioned above, many components participate in IL-6 signaling system. It enlarges the spectrum of IL-6 target cells because cells which do not express a mIL-6R can be stimulated by IL-6 and sIL-6R. Moreover, since hepatocytes express far more gp130 than mIL-6R, it has been shown that IL-6/sIL-6R has more effective on hepatocytes than IL-6 alone [14, 15]. 

## 3. IL-6 and Soluble IL-6 Receptor in RA Patients

Overproduction of IL-6 has been found in the synovial fluid and blood of RA patients, and IL-6 levels correlate with disease activity [16, 17]. On the other hand, sIL-6R is present in the blood of both healthy subjects and RA patients, and the concentration is comparable between healthy subjects and RA patients. In contrast, a higher concentration of sIL-6R is detectable in the synovial fluid of RA patients than is found in the synovial fluid of osteoarthritis patients [18]. Inflammatory cells such as monocytes and lymphocytes infiltrating into the synovium are considered to be a source of sIL-6R. 

## 4. Roles of IL-6 in Synovitis

Changes in the synovium are marked by neovascularization, infiltration of inflammatory cells, and synoviocyte hyperplasia that act together to produce a pannus (inflammatory vascular tissue). Newly formed blood vessels are thought to be involved in the development and maintenance of synovitis because they support the infiltration of inflammatory cells and the growth and survival of synovial cells.

Although a number of growth factors and cytokines have angiogenic activity, vascular endothelial growth factor (VEGF) is thought to be the most important angiogenic factor in the pathogenesis of RA [19]. Significant increases in VEGF levels in RA patients correlate with disease activity, suggesting that VEGF is implicated in RA pathogenesis, particularly in pannus formation. In RA patients, VEGF levels in blood are elevated and treatment with TCZ significantly lowers VEGF levels [20]. IL-6 induced tubule formation in a coculture system of human umbilical venous endothelial cells (HUVECs) and fibroblast-like synoviocytes from RA patients (RA-FLS) and that this angiogenesis was completely inhibited by anti-VEGF antibody, indicating that VEGF plays a crucial role in IL-6-induced angiogenesis [21].

TCZ treatment significantly reduced joint swelling and the infiltration of inflammatory cells into inflamed joints in monkey collagen-induced arthritis (CIA), when TCZ was injected after the onset of arthritis [22]. IL-6 augmented production of chemokines such as monocyte chemotactic protein-1 (MCP-1) and IL-8 from endothelial cells, mononuclear cells, and RA-FLS, and also induced adhesion molecules such as ICAM-1 in endothelial cells and increased adhesion of monocytes to endothelial cells [23, 24]. These lines of evidence strongly support the idea that IL-6 aggravates the local inflammatory reaction by amplifying inflammatory cell infiltration. Suppression of angiogenesis may also reduce cell migration, because newly formed blood vessels are conduits for the infiltration of inflammatory cells.

Synovial fibroblastic cells produced large amounts of IL-6 when stimulated by inflammatory cytokines such as IL-1, TNF*α*, and IL-17, and that IL-6 augmented the proliferation of synovial fibroblastic cells in the presence of sIL-6R [25, 26]. TCZ may exert its antisynovitis effect via the inhibition of these biological activities of IL-6. In fact, semiquantitative ultrasonographic assessment clearly indicates that TCZ treatment significantly improves synovitis in RA patients [27]. 

## 5. Roles of IL-6 in Joint Damage

Irreversible joint destruction is a characteristic feature of RA. TCZ monotherapy for 52 weeks showed significantly less radiographic change in total Sharp score (bone erosion and joint space narrowing) than DMARD treatment [28].

**Figure 2:**
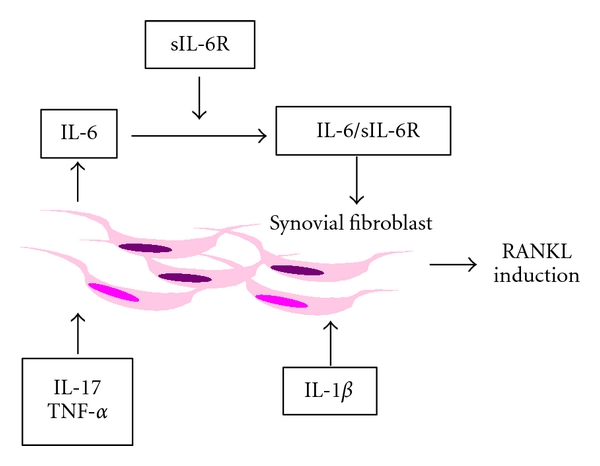
Mechanism of RANKL induction by cytokines.

As a pathogenic mechanism of bone destruction, osteoclasts activated by inflammatory cytokines are thought to be responsible for focal bone erosion. Indeed, osteoclasts are often seen in the synovium at sites of cartilage destruction in RA patients [29, 30]. The receptor activator of NF-*κ*B (RANK) and its ligand (RANKL) are essential factors for osteoclastogenesis [31–33]. IL-6 and sIL-6R, but not IL-6 alone, induced RANKL expression in RA-FLS. On the other hand, TNF*α* and IL-17 did not induce RANKL expression, although both stimulate cell growth and IL-6 production. Interestingly, in the presence of sIL-6R, TNF*α* or IL-17 induced RANKL expression (Figure 2). In a coculture of RA-FLS and osteoclast precursor cells, IL-6 and sIL-6R induced NFATc1 and TRAP5b mRNA expression in the osteoclast precursor cells. IL-6/sIL-6R directly induced osteoclastogenesis by inducing RANKL expression in RA-FLS [26]. Moreover, in mouse calvarial bone cultures, IL-6, in the presence of sIL-6R, induced bone resorption, which was decreased by osteoclast inhibitors, suggesting that IL-6 signaling influences osteoclastogenesis induced by osteoblast and osteoclast interaction [34]. From these facts, IL-6 induces osteoclast formation by inducing RANKL in RA-FLS as well as osteoblast.

Cartilage degeneration is also observed in RA joints. RANKL inhibition clearly halted the progression of bone erosion, but did not improve joint space narrowing in RA patients, strongly suggesting that RANKL/RANK signaling does not participate in cartilage degeneration in RA patients [35]. Matrix metalloproteinases (MMPs) and a disintegrin and metalloproteinase with thrombospondin-like repeat (ADAMTSs) are thought to play crucial roles in cartilage matrix degeneration. IL-6 induced MMP-1, MMP-3, and MMP-13 production from chondrocytes and synovial cells [36, 37]. On the other hand, it is reported that tissue inhibitors of MMPs (TIMPs) are endogenous inhibitors of MMPs. IL-6, in the presence of sIL-6R, induced the production of TIMP in cultured human chondrocytes and synovial fibroblasts, suggesting that IL-6 plays a role in extracellular matrix turnover [38].

These data suggest that the preventive effect of TCZ on joint destruction is mediated by the inhibition of IL-6-induced RANKL induction followed by osteoclastogenesis and suppression of the IL-6-induced production of MMPs. In the SAMURAI and OPTION studies, improvement in a bone resorption marker (C-terminal cross-linking telopeptide of type I collagen) and in cartilage turnover markers (N-terminal propeptide of type IIA collagen and type II collagen helical peptide) were seen in the TCZ group [28, 39]. Moreover, tocilizumab decreased serum MMP-3 levels in several clinical trials [40–42]. 

## 6. Roles in Autoimmunity

There is no doubt that T cells play important roles in the onset of RA. CD4^+^T helper cells have been classified as T_h_1 and T_h_2 cells on the basis of their cytokine production profiles, and, recently, T_h_17 cells which produce IL-17 in autoimmune pathology have become recognized as a separate subset, which is interesting in the context of events that were previously thought to be T_h_1-mediated. In vitro studies in mice have shown that the costimulation of IL-6 and TGF-*β* is essential for the differentiation of T_h_17 cells from naïve CD4^+^ T cells [43]. Furthermore, studies suggesting an involvement of IL-6 in the induction of T_h_17 cells in arthritis models have been reported: anti-IL-6R antibody suppressed the onset of arthritis in G6PI-induced and collagen-induced arthritis models and concomitantly inhibited the appearance of T_h_17 cells [44, 45]. The involvement of T_h_17 cells in RA is, therefore, still controversial. However, the involvement of CD4^+^CD161^+^ T cells in autoimmune diseases has been attracting attention recently [46]. These T cells produce large amounts of IL-17 and are increased in psoriasis and Crohn's disease. It is also reported that peripheral blood mononuclear cells (PBMC) from RA patients produced higher levels of IL-17 than PBMC from healthy subjects when stimulated with anti-CD3 and anti-CD28 antibodies [47]. The role of IL-17 producing T cells in RA will be clarified in the near future.

Moreover, RA is characterized by an increase in IgM and IgG rheumatoid factors and antibodies to citrullinated peptides in both serum and joints. B-cell depletion is of therapeutic benefit in RA and demonstrates the impact of B-cell activity on synovial inflammation and joint damage in this disease. IL-6 was originally identified as a B-cell differentiation factor; it plays an important role in the development of antibody-producing plasma B cells [48]. IL-6 induces B-cell differentiation through its action on plasmablasts [49] and more recently has been shown to induce B-cell antibody production indirectly by promoting the B-cell helper properties of CD4^+^ T cells via the production of IL-21 [50]. Indeed, TCZ treatment decreased frequency of circulating plasma cells in SLE patients [51]. 

## 7. Roles of IL-6 in Anemia of RA

Anemia is the most common extra-articular manifestation of RA and is estimated to occur in 30% to 60% of patients [52, 53]. There are two primary types of anemia in RA: anemia of chronic disease (ACD) and iron-deficiency anemia. ACD is characterized by hypoferremia in the presence of adequate iron stores. ACD is an inflammatory anemia, and inflammatory cytokines are thought to play important roles in anemia in RA [54, 55]. In fact, TCZ therapy in RA patients rapidly improves anemia [40].

Anemia was also induced in monkey CIA after collagen immunization. Anemia in monkeys with CIA is characterized by decreased serum iron and transferrin (Tf)-saturation, and by elevated serum ferritin, and its severity is correlated with serum IL-6 levels; therefore, anemia in monkeys with CIA is very similar to human anemia in inflammatory diseases, at least with respect to the changes in serum parameters [7]. Hepcidin is a master regulator of iron homeostasis in humans and other mammals [56]. It inhibits the absorption of iron in the small intestine and the release of recycled iron from macrophages, effectively decreasing the delivery of iron to maturing erythrocytes in the bone marrow [8]. In fact, mice genetically engineered to overproduce hepcidin die of severe iron deficiency shortly after birth [57]. Interestingly, IL-6 induces hepcidin production in liver cells [58].

**Figure 3:**
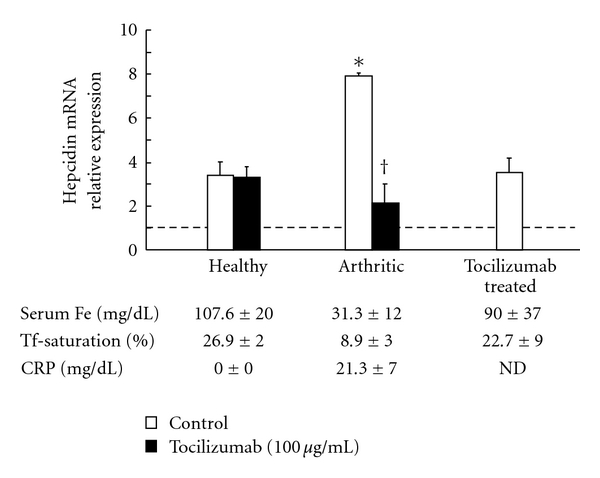
Hepcidin mRNA induction by arthritic serum in Hep3B cells [7]. Hep3B cells were incubated with serum from healthy, arthritic, or tocilizumab-treated monkeys for 24 h. Tocilizumab was added simultaneously with serum. After incubation, total mRNA was extracted, and hepcidin mRNA was measured by real-time PCR. The hepcidin expression induced by medium alone (without serum) was defined as 1. Each point represents the mean and SD of 3 monkeys. Statistical significances between healthy animals and arthritic animals and between control and the addition of tocilizumab were analyzed by unpaired *t*-test. *P < .05 (healthy animals' serum, control versus arthritic animals' serum, control), ^†^ *P* < .05 (arthritic animals' serum, control versus arthritic animals' serum, tocilizumab).

Administration of TCZ to monkeys with CIA rapidly improved anemia and induced a rapid but transient reduction in serum hepcidin. Hepcidin mRNA expression was more potently induced by serum from arthritic monkeys, and this was inhibited by the addition of TCZ (Figure 3) [59]. From these lines of evidence, we propose that TCZ improves anemia in monkey arthritis via the inhibition of IL-6-induced hepcidin production. 

## 8. Roles of IL-6 in Hypolipidemia

**Figure 4:**
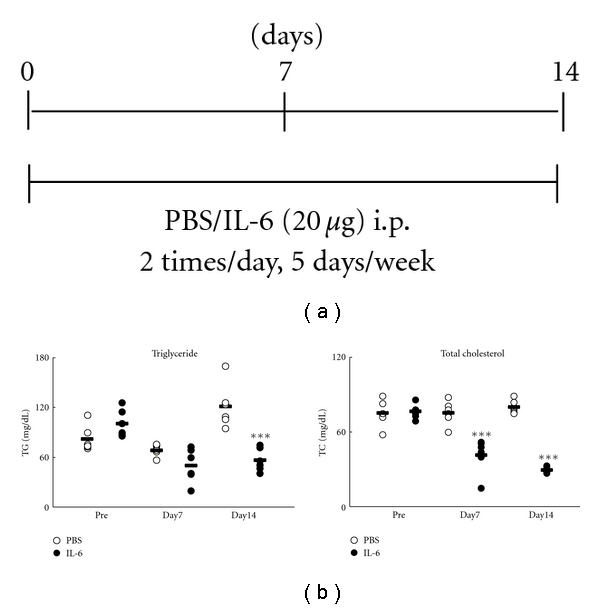
Serum lipid levels in IL-6-treated mice [8]. (a) Experimental protocol. Mice (*n* = 6) were given i.p. IL-6 (20 *μ*g) or phosphate buffered saline (PBS) twice a day 5 days per week for 2 weeks. (b) Serum total cholesterol and triglyceride levels were measured with an automatic analyzer. Closed and open circles indicate control and IL-6-treated mice, respectively. The horizontal bar indicates mean of values. Statistical significance between the control and the IL-6 group on days 0, 7, and 14 was analyzed by unpaired *t*-test (***P < .05).

Cholesterol and triglyceride levels appear normal or even low in patients with early active RA and high-grade inflammation [60]. We previously reported that IL-6-treated mice had low total cholesterol (TC) and triglyceride (TG) levels compared with PBS-treated mice (Figure 4) [61]. In this model, we confirmed that expression of the VLDL receptor, which plays a role in the delivery of fatty acids derived from VLDL-triglycerides from the blood to peripheral tissues, was upregulated in IL-6-treated mice. From these results, it is suggested that the induction of VLDL receptor by IL-6 may be related to the hypolipidemia. Several reports have described the function of IL-6 in lipid metabolism in adipose tissue. Interstitial IL-6 concentrations in adipose tissue are ~100-fold higher than in plasma, implying an important auto- and paracrine regulatory function in this tissue [62]. IL-6 has lipolytic properties and increases lypolysis of adipose tissue and adipocytes in vitro [63, 64]. Consistent with these in vitro studies, IL-6 infusion in humans increased free fatty acid and whole body fat oxidation [65].

It is reported that treatment of RA patients with TCZ increased blood levels of TC, TG, and HDL-cholesterol in a manner inversely related to the disease activity of RA [66]. It is also reported that blockade of TNF*α* increased blood levels of TC, TG, and HDL-cholesterol, and that the persistent inflammatory condition reflected by elevated serum TNF*α* levels results in low levels of TC and TG in RA [67–69]. This fact strongly suggests that the inhibition of IL-6 production induced by TNF*α* blockade results in an increase of lipids. Anyway, both TNF*α* blockade and TCZ did not change the atherogenic index (TC/HDL) although these drugs increased TC and TG [70, 71]. 

## 9. Conclusion

**Figure 5:**
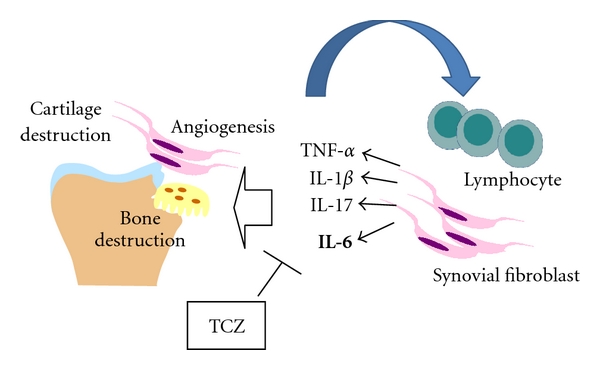
Mode of action of tocilizumab.

IL-6 is considered to play a central role in chronic inflammation and is expressed in excess at sites of inflammation. IL-6 levels are considerably elevated in the serum of RA patients, and this elevation has been directly correlated with clinical indices of disease activity. In addition, high levels of sIL-6R have been shown to correlate with the degree of joint destruction, in particular, in advanced stages of RA. IL-6 is a multitarget cytokine with activity relevant to RA. At the affected joints, IL-6 has a pivotal role in the inflammatory process, in osteoclast-mediated bone resorption, and in synovitis (Figure 5). IL-6 induces acute-phase proteins and contributes to the systemic manifestations of RA though hepcidin production (anemia) and acts potently in changing lipid concentrations (hypolipidemia). In addition, IL-6 may contribute to the induction and maintenance of the autoimmunity through B-cell activation and T_h_17 cell differentiation.

TCZ has been recently approved for the treatment of adult patients with moderately to severely active RA with inadequate response to one or more DMARDs or TNF antagonists. TCZ not only improves local signs and symptoms, but also systemic ones, such as anemia, anorexia, fever, and fatigue, thereby potentially improving patient QOL. Infection is among the most common adverse effect of cytokine inhibitors. Although the incidence of infections is similar to that with other biologics and most episodes are not serious and can be straightforwardly managed, TCZ significantly reduces inflammatory markers such as CRP. When administering TCZ, it is important to pay attention not only to abnormal test values associated with infection but also to the emergence of symptoms of infection in the patients. It is also important to educate patients who are going to be treated with TCZ that they should come in promptly for treatment if they notice any physical abnormalities, such as cough or sputum. 

In conclusion, IL-6 participates in both the inflammation and autoimmunity of RA patients. Therefore, the blockade of IL-6 is a beneficial therapy for RA patients.
